# Specialized Metabolites in Cereal Crops Under Abiotic Stress: Regulatory Mechanisms, Functional Validation, and Breeding Implications

**DOI:** 10.3390/plants15192905

**Published:** 2026-09-23

**Authors:** Muhammad Ali Shah, Imran Azeem, Rehmat Ullah, Haseeb Ahmad, Waqas Liaqat, Muhammad Faheem Jan

**Affiliations:** 1 School of Breeding and Multiplication (Sanya Institute of Breeding and Multiplication), Hainan University, Sanya 572025, China; 2 Key Laboratory of Crop Genetic Breeding and Germplasm Innovation, Guangxi Key Laboratory of Agro-Environment and Agri-Products Safety, College of Agriculture, Guangxi University, Nanning 530004, China; 3 Guangxi Key Laboratory of Agro-Environment and Agro-Products Safety, College of Agriculture, Guangxi University, Nanning 530004, China; 4 Department of Field Crops, Faculty of Agriculture, Recep Tayyip Erdoğan University, Rize 53300, Türkiye

**Keywords:** cereals, abiotic stress, flavonoids, phenylpropanoid, lignin, carotenoids, metabolomics, transcription factors, hormonal crosstalk, metabolic engineering

## Abstract

Cereal crops increasingly encounter drought, salinity and alkalinity, heat, waterlogging, potentially toxic elements, and compound stresses that constrain productivity and grain quality. Secondary metabolites, also referred to as specialized metabolites in the recent literature, represent a dynamic component of cereal stress adaptation rather than a passive catalogue of stress markers. This review aims to synthesize current evidence on how specialized-metabolite pathways contribute to abiotic stress responses in cereals, with particular emphasis on regulatory mechanisms, evidence strength, chemical form, subcellular localization, recovery, and translational relevance. We critically assess evidence from transcriptomics, metabolomics, genetic perturbation, biochemical assays, multi-omics integration, and physiological studies across major cereal crops and evaluate evidence according to causal, associative, and in vitro categories. The evidence reveals that perturbations at the plasma membrane, cell wall, chloroplast, mitochondrion, and endomembrane system levels generate reactive oxygen species, Ca^2+^ signatures, phospholipid signals, and hormone changes that are decoded by CDPK/CPK, CBL–CIPK, SnRK2, and MAPK cascades. These signaling networks converge on MYB–bHLH–WD40, WRKY, NAC, ERF/AP2, HSF, and bZIP regulators and redirect carbon and reducing power through phenylpropanoid, flavonoid, lignin, carotenoid, terpenoid, benzoxazinoid, cyanogenic, and related pathways. Comparative analysis across rice (*Oryza sativa* L.), wheat (*Triticum aestivum* L.), maize (*Zea mays* L.), barley (*Hordeum vulgare* L.), oat (*Avena sativa* L.), sorghum (*Sorghum bicolor* L.), and millets shows conserved regulatory features but substantial crop-, genotype-, tissue-, developmental-stage-, and dose-dependent variation. Functional evidence further indicates that enzyme activity, chemical modification, transport, and subcellular compartmentation can determine whether metabolites contribute to ROS buffering, photosynthetic and membrane protection, cell-wall reinforcement, osmotic or ionic homeostasis, toxic-ion sequestration, or signaling. The review also identifies important limitations in current research, including overreliance on associative omics evidence, insufficient consideration of combined stresses and rehydration, growth–defence trade-offs, and limited field and reproductive-stage validation. To distinguish growth dilution from genuine biosynthetic increases, absolute metabolite content per grain or organ should be measured alongside concentration per unit dry weight. We conclude that improving cereal resilience requires context-dependent and experimentally validated coordination of regulators, biosynthetic enzymes, chemical modification, transport, and compartmentation rather than indiscriminate elevation of total specialized-metabolite concentration.

## 1. Introduction

Cereal crops underpin global food and nutritional security, yet their productivity and grain quality are increasingly threatened by drought, salinity and alkalinity, extreme heat, transient flooding, soil oxygen deficiency, and contamination by potentially toxic elements. The global scale of these losses indicates the urgency of improving cereal resilience: the Food and Agriculture Organization estimated that disasters caused cumulative losses of approximately 4.6 billion tonnes of cereals between 1991 and 2023, highlighting the substantial production burden imposed by increasingly frequent and severe agricultural shocks [1]. Anthropogenic climate change has already constrained agricultural productivity, while crop modelling projects increasingly severe effects across major cereal-producing regions [2,3]. Under a high-emissions RCP8.5 scenario, recent global projections accounting for observed adaptation estimate end-of-century yield losses of approximately 28% for maize, 28% for wheat, and 6% for rice, although substantial uncertainty remains across crops and regions [4]. At the cellular level, these stresses disturb water and ion homeostasis, membrane integrity, electron transport, protein stability, and carbon allocation, thereby altering redox balance, energy metabolism, and source–sink relations. At the ultrastructural level, these disturbances can cause plasma-membrane disorganization, chloroplast swelling and thylakoid disruption, mitochondrial structural damage, vacuolar alterations, and cell-wall changes, ultimately impairing cellular function, reproductive development, and grain filling [5]. Crop performance therefore depends on the balance among cellular injury, stress signaling, metabolic adjustment, recovery, and the resource costs of defence. These stresses can redirect carbon and nitrogen allocation, alter carbohydrate and amino-acid metabolism, increase or deplete organic acids and osmoprotectants, modify lipid turnover, and stimulate antioxidant and specialized-metabolite pathways to maintain redox and osmotic homeostasis. Secondary metabolites (also referred to as specialized metabolites in the recent literature) occupy a central position within this balance. These specialized metabolites influence cellular stress responses through distinct metabolic and biochemical processes. Phenylpropanoids and flavonoids can scavenge reactive oxygen species and modulate redox signaling, while anthocyanins and carotenoids absorb excess light and help protect photosynthetic membranes. Lignin precursors are polymerized in the cell wall to strengthen structural barriers and restrict uncontrolled ion and water movement, whereas terpenoids can function as signaling molecules or membrane-associated compounds. Benzoxazinoids and cyanogenic glycosides provide chemically stored defense capacity that can be activated through tissue damage or stress-induced turnover and can also influence interactions with herbivores and soil microorganisms [6,7]. They also influence grain quality through specific bioactive compounds: anthocyanins and carotenoids contribute to grain pigmentation and antioxidant properties; phenolic acids, flavonoids, and tocopherols influence nutritional and oxidative-stability traits; phenolic compounds and lignin-related components can affect processing characteristics; and benzoxazinoids, alkaloids, and cyanogenic glycosides can influence palatability and food or feed safety through their biological activities and, in some cases, antinutritional or toxic effects. Their biological effects, however, depend on chemical structure, concentration, tissue, developmental stage, subcellular location, and stress context.

The conventional simple binary separation between primary and specialized metabolism can obscure the extensive metabolic crosstalk that occurs during stress. Phenylpropanoid and isoprenoid pathways draw carbon skeletons, energy, reducing equivalents, and precursors from central metabolism; consequently, stress-induced activation of specialized-metabolite pathways can alter resource allocation between defence, growth, and reproduction [8,9]. This resource-allocation perspective helps explain why enhanced specialized-metabolite production may improve stress protection in some contexts but may also impose costs on biomass accumulation, reproductive development, or grain filling. These pathways consume carbon skeletons, aromatic amino acids, reducing equivalents, activated sugars, and methyl donors that also support growth and reproduction. Stress-induced accumulation may therefore indicate successful acclimation, but it may also reflect growth inhibition, reduced biomass dilution, impaired transport, slower degradation, tissue injury, or diversion of resources from grain formation. Consequently, a response that increases survival but suppresses root growth, flowering, or grain filling may have limited agronomic value [10]. Chemical form and cellular localization further determine metabolite function. Glycosylation, methylation, hydroxylation, and acylation alter solubility, reactivity, stability, transport, and sequestration. Flavonoids comprise structurally diverse subclasses, including flavonols, flavones, flavanones, and anthocyanins, whose cellular functions depend on their chemical form and localization. Flavonols and related flavonoids can interact with reactive oxygen species and redox-sensitive processes near chloroplasts, nuclei, and membranes, thereby potentially modulating oxidative signaling and membrane protection. Anthocyanins are predominantly glycosylated and stored in vacuoles, where they contribute to light screening, pigmentation, and antioxidant capacity. Glycosylation and other chemical modifications influence their solubility, stability, transport, and subcellular sequestration and therefore determine their accessibility and biological activity under stress [11]. Genetic manipulation has shown that flavonoid accumulation can improve oxidative and drought tolerance, although evidence from *Arabidopsis thaliana* cannot be extrapolated directly to cereals [12]. Mechanistic interpretation must therefore move beyond lists of differentially accumulated compounds towards pathway flux, chemical identity, transport, compartmentation, and physiological consequence.

Cross-cereal synthesis remains difficult because metabolomics studies differ in extraction chemistry, chromatography, ionization, quantification, spectral libraries, and annotation confidence. A reported flavonoid may represent an authentic-standard-confirmed compound, a high-confidence tandem mass spectrometry annotation, a tentative molecular formula, or only a pathway-level assignment, and these levels of annotation confidence cannot support equivalent biological conclusions [13]. Experimental conditions also require careful distinction. Polyethylene glycol-induced osmotic stress does not reproduce all features of progressive soil drought, sodium chloride exposure does not represent every saline–alkaline soil, and short heat shocks differ from chronic heat during reproductive development [14]. Likewise, root-zone hypoxia, waterlogging, complete submergence, and reoxygenation impose distinct metabolic constraints [15]. These differences also complicate the interpretation of mechanistic evidence because coordinated transcript–metabolite changes may identify candidate pathways without establishing causal relationships. Accordingly, evidence strength must be distinguished according to the type of experimental support available, as detailed in the Evidence Assessment Framework (Section 3.5). Translation is also restricted by the dominance of controlled experiments conducted at early developmental stages with limited genotypic diversity. Cereal improvement requires validation during reproductive development, recovery, and variable field conditions, with yield, grain quality, and growth defence trade-offs considered explicitly [16,17].

The objective of this review is to evaluate the mechanistic evidence linking specialized-metabolite regulation with abiotic stress responses in major cereal crops, while identifying key knowledge gaps and translational priorities for developing stress-resilient and agronomically productive cereals. This review therefore organizes cereal specialized metabolism along a mechanistic chain linking stress perception, reactive oxygen species and Ca^2+^ signaling, kinase and hormone crosstalk, transcriptional regulation, pathway activity, chemical tailoring, transport, compartmentation, cellular protection, recovery, and agronomic performance. However, an important gap remains between the increasing number of studies reporting stress-responsive metabolites and a mechanistic understanding of their functions in cereal stress resilience. Many studies rely primarily on transcriptomic or metabolomic associations, with less emphasis on causal evidence, chemical form, subcellular localization, recovery, and agronomic performance. This review addresses these gaps by integrating mechanistic evidence with physiological and translational perspectives. It covers major cereal species, including rice (*Oryza sativa*), wheat (*Triticum aestivum*), maize (*Zea mays*), barley (*Hordeum vulgare*), oat (*Avena sativa*), sorghum (*Sorghum bicolor*), and representative millets, exposed to drought, salinity and alkalinity, heat, waterlogging and hypoxia, potentially toxic elements, and combined stresses. The central proposition is that no universal ranking of “beneficial metabolites” is defensible. Translationally useful targets are context-defined regulatory modules specified by crop, genotype, organ, developmental stage, stress intensity, chemical form, subcellular location, and strength and type of supporting evidence, as evaluated through the framework in Section 3.5.

## 2. Materials and Methods

### 2.1. Review Scope and Literature Search

This review critically evaluated the roles of specialized metabolites in abiotic-stress responses of major cereal crops, including rice, wheat, maize, barley, oat, sorghum, and millets. The relevant literature was identified using the Web of Science Core Collection, Scopus, and PubMed, with Google Scholar used for supplementary citation tracking. The primary publication coverage extended from January 2001 to August 2026. Earlier seminal studies were retained selectively when they provided essential mechanistic context, including the foundational work of Dixon and Paiva [8] on stress-induced phenylpropanoid metabolism. Search terms were organized into four groups covering cereal crops, abiotic stresses, specialized metabolites, and mechanistic or analytical evidence. Representative terms included rice, wheat, maize, barley, sorghum, drought, rehydration, salinity, alkalinity, heat stress, waterlogging, hypoxia, cadmium, arsenic, flavonoids, phenylpropanoids, lignin, carotenoids, metabolomics, transcriptomics, CRISPR, gene expression, enzymes, transporters, metabolic flux, and recovery. Terms within groups were combined using OR, whereas the major concept groups were combined using AND.

### 2.2. Study Selection and Information Extraction

Peer-reviewed original studies were prioritized when they linked abiotic stress with specialized-metabolite regulation, accumulation, modification, transport, localization, or function in cereals. Titles and abstracts were screened first, followed by full-text assessment of potentially relevant studies. Studies were excluded when they lacked a relevant abiotic-stress component, did not address specialized metabolism, focused exclusively on biotic stress, or provided insufficient mechanistic information. For eligible studies, information was extracted on crop and genotype, stress treatment, tissue or developmental stage, analytical platform, metabolite class, associated genes or regulators, and physiological or agronomic outcomes, including recovery, yield, grain quality, and safety where available.

### 2.3. Assessment of Mechanistic Evidence

Evidence was classified as causal, associative, or in vitro. Causal evidence included genetic or functional perturbation, such as knockout, gene silencing, overexpression, complementation, rescue, transporter manipulation, or related validation approaches [18]. Associative evidence included transcriptomic or metabolomic correlations, genotype comparisons, pathway enrichment, and related observational relationships. In vitro evidence included enzyme assays, purified-protein analyses, promoter-binding assays, and other biochemical tests performed outside the intact plant. Metabolite-identification and quantification confidence were also considered during interpretation, particularly where conclusions depended on metabolomics-based evidence [13,19].

### 2.4. Evidence Synthesis

Evidence was synthesized across crop species, stress type, metabolite class, tissue, developmental stage, mechanistic support, and physiological or agronomic outcome. Increased metabolite abundance alone was not considered sufficient evidence of adaptive function; greater weight was given to studies showing convergence of molecular, chemical, physiological, and agronomic responses. The overall literature-search, screening, evidence-classification, and synthesis procedure is summarized in Figure 1.

## 3. Molecular Sensing, Signaling, and Transcriptional Control

Abiotic stress signaling in cereals operates as a layered network rather than a linear pathway [5]. Perturbations at the plasma membrane, cell wall, chloroplast, mitochondrion, and endomembrane system generate reactive oxygen species (ROS), Ca^2+^, nitric oxide, and lipid-derived signals [20]. Kinase and hormone networks decode these signals and redirect transcription, enzyme activity, transport, and metabolic flux. Although different stresses recruit overlapping regulatory nodes, pathway output depends on signal origin, intensity, duration, tissue, developmental stage, and recovery status. Secondary-metabolite accumulation may therefore support acclimation, reflect cellular injury, or represent both processes simultaneously.

### 3.1. From Stress Perception to Second-Messenger Signatures

Abiotic stresses initially disturb cellular physical or chemical homeostasis. Drought changes turgor, membrane tension, and cell wall mechanics; salinity combines osmotic stress with Na^+^ and Cl^−^ toxicity; heat disrupts membrane fluidity and protein stability; waterlogging restricts oxygen supply and mitochondrial respiration; and potentially toxic elements alter metal and redox homeostasis [5]. Receptor-like kinases, mechanosensitive channels, osmosensitive channels, and organelles redox systems convert these disturbances into intracellular signals. For example, OSCA1 forms a hyperosmolality-gated Ca^2+^-permeable channel required for normal osmotic signaling in *Arabidopsis thaliana*, although equivalent functions require direct validation in cereals [21].

Ca^2+^ and ROS interact through reciprocal feedback. Ca^2+^-dependent protein kinases can phosphorylate respiratory burst oxidase homologues, promoting apoplastic ROS production, whereas ROS can alter Ca^2+^-channel activity and organelle permeability [20]. Chloroplast ROS primarily indicate photochemical imbalance, mitochondrial ROS reflect respiratory disruption, and peroxisomal H_2_O_2_ links photorespiration and fatty-acid metabolism with cellular redox regulation. Aquaporins can also facilitate regulated H_2_O_2_ movement across membranes, allowing local oxidative events to influence cytosolic and nuclear signaling.

Signal identity depends on amplitude, frequency, duration, and subcellular origin rather than on total Ca^2+^ or ROS concentration alone [22]. Productive acclimation therefore requires controlled ROS production, transport, sensing, and scavenging. Insufficient buffering promotes lipid peroxidation, protein oxidation, and cell death, whereas excessive scavenging may suppress essential redox signals.

### 3.2. Kinase Relays and Hormone Integration

Ca^2+^ signatures are decoded by calmodulins, Ca^2+^-dependent protein kinases, and calcineurin B-like protein–CBL-interacting protein kinase complexes. These modules regulate ion transporters, respiratory burst oxidases, transcription factors, and metabolic enzymes. Mitogen-activated protein kinase cascades integrate receptor, redox, and hormone inputs, whereas SNF1-related protein kinase 2 proteins connect osmotic stress with abscisic acid (ABA) signaling [5].

In the core ABA pathway, ABA binding to PYRABACTIN RESISTANCE/PYR1-LIKE receptors inhibits clade A protein phosphatase 2Cs and releases SNF1-related protein kinase 2 activity. This phosphorylation network regulates stomatal behavior, ion channels, osmotic adjustment, and stress-responsive transcription [23]. Jasmonate links lipid-derived signaling with phenylpropanoid and terpenoid metabolism, while ethylene contributes strongly to flooding and hypoxia responses. Salicylic acid, auxin, and brassinosteroids further modify redox regulation and growth-defense allocation. These hormones function through context-dependent crosstalk rather than as independent switches; their effects vary among tissues, developmental stages, and stress sequences [24].

### 3.3. Transcription-Factor Complexes and Pathway Branch Points

Transcription factors connect kinase and hormone signaling with secondary-metabolite biosynthesis. MYB, basic helix–loop–helix, and WD40 proteins form regulatory complexes that control flavonoid, anthocyanin, and proanthocyanidin pathway genes [25]. WRKY factors connect ROS and hormone signals with phenylpropanoid defense, whereas NAC factors regulate secondary-wall formation, lignification, and senescence. APETALA2/ethylene-responsive factors contribute to hypoxia responses, while heat-shock factors coordinate thermal protection with antioxidant and metabolic regulation. These regulators can activate one pathway branch while repressing another. Their effects depend on promoter context, interacting proteins, tissue identity, and developmental stage. Chromatin accessibility, DNA methylation, histone modification, small RNAs, protein turnover, and metabolite feedback further determine whether transcriptional induction produces sustained pathway flux [24]. Transcript abundance alone is therefore insufficient evidence that a biosynthetic pathway has become functionally active.

### 3.4. Enzyme Complexes, Transport, and Compartmentation

Secondary-metabolite pathways are spatially organized. Phenylalanine ammonia-lyase operates mainly in the cytosol, whereas cinnamate 4-hydroxylase and associated enzymes are linked with the endoplasmic reticulum. Interacting enzymes can form metabolons that channel reactive intermediates towards specific products and reduce competition among pathway branches. Direct cereal evidence comes from sorghum, in which membrane-associated cytochrome P450 enzymes and a soluble glucosyltransferase assemble into a dynamic metabolon for dhurrin biosynthesis [26]. Carotenoid synthesis occurs within plastids, where enzyme localization, plastid development, sequestration capacity, and photosynthetic demand regulate accumulation [27]. Chemical tailoring and transport provide an additional regulatory layer. Glycosyltransferases, methyltransferases, hydroxylases, and acyltransferases modify metabolite solubility, reactivity, stability, and recognition. Glutathione *S*-transferase ligandins, multidrug and toxic compound extrusion transporters, ATP-binding cassette transporters, and vesicular trafficking contribute to intracellular transport and vacuolar sequestration [28]. Biosynthesis, storage, and biological activity may therefore occur in different compartments. A vacuolar anthocyanin, plastidial carotenoid, membrane-associated flavonols, and wall-bound hydroxycinnamates cannot be assigned the same function from total concentration alone [7].

### 3.5. Evidence Assessment Framework: Mechanistic Integration and Evidence Strength

Evidence for secondary-metabolite function should be evaluated according to the strength and type of supporting evidence rather than inferred from metabolite abundance or pathway enrichment alone. We propose a three-tier evidence framework comprising causal, associative, and in vitro evidence. Causal evidence provides the strongest support for biological function and includes experimental evidence demonstrating that manipulation of a gene, enzyme, transporter, metabolite, or pathway alters the stress-response phenotype and that restoration or targeted chemical intervention can reverse or reproduce the effect. Associative evidence includes pathway enrichment, coordinated transcript–metabolite changes, temporal concordance, allele contrasts, quantitative trait locus (QTL) colocalization, and other correlative relationships that support biological association but do not, by themselves, establish causality. In vitro evidence, including enzyme activity assays, purified-protein analyses, promoter-binding assays, and biochemical validation outside the intact plant, can provide mechanistic support but should be distinguished from evidence demonstrating physiological function in planta. Three principles are particularly important when assessing evidence for secondary-metabolite function. First, correlation does not establish causation: changes in metabolite abundance or transcript expression may accompany stress responses without being responsible for the observed phenotype. Second, total metabolite concentration does not necessarily reflect biological function because activity may depend on tissue distribution, cellular compartmentation, chemical form, bioavailability, and interaction with specific molecular targets. Third, chemical modification and subcellular localization can substantially alter metabolite activity, stability, transport, and accessibility, meaning that measurements of total abundance alone may not adequately represent functional significance. Causal inference should therefore be strengthened by complementary approaches, including gene perturbation, promoter-binding assays, enzyme validation, isotope tracing, transporter analysis, spatial localization, and chemical rescue, which can provide evidence for necessity, sufficiency, biochemical activity, or pathway connectivity [18]. However, the interpretation of metabolite abundance requires additional caution because observed changes may result from altered synthesis, degradation, transport, sequestration, tissue composition, or growth dilution. Metabolite identification and quantification confidence should also be established before biological function is assigned [13]. These evidence levels should be considered within an integrated regulatory architecture in which stress perception, kinase and hormone signaling, transcriptional regulation, biosynthesis, chemical tailoring, transport, and compartmentation connect stress exposure with specialized-metabolite function (Figure 2). The principal sensing, regulatory, biosynthetic, tailoring, and transport modules, together with their subcellular locations, proposed functions, and levels of supporting evidence, are summarized in Table 1. Throughout Section 3, Section 4, Section 5, Section 6, Section 7, Section 8, Section 9, Section 10 and Section 11, evidence is interpreted according to this framework, and individual studies are classified as causal, associative, or in vitro where the available evidence permits.

## 4. Drought, Rehydration, and Secondary Metabolite Allocation

Drought redirects carbon and reducing power among growth, osmotic adjustment, antioxidant defense, and specialized metabolism. Comparative cereal studies commonly associate tolerant genotypes with stronger flavonoid and phenylpropanoid responses, but the strength of evidence supporting a protective role should be evaluated according to the Evidence Assessment Framework defined in Section 3.5. Direct evidence is provided by *OsCHI3* in rice: overexpression increased flavonoid accumulation and drought tolerance, whereas CRISPR/Cas9 knockout increased drought sensitivity; exogenous abscisic acid partly rescued the mutant phenotype [46]. This regulatory module connected flavonoid metabolism with reactive oxygen species scavenging and abscisic acid biosynthesis, thereby linking gene function, chemical reprogramming, and stress performance. Field evidence demonstrates why metabolite accumulation must be interpreted alongside injury and yield. In barley, drought increased several phenolic acids, flavonoids, hydrogen peroxide, malondialdehyde, and antioxidant activities, while photosynthetic performance and grain yield declined [47]. High phenolic or antioxidant activity may therefore accompany either successful acclimation or persistent oxidative stress. Lignin and wall-bound phenolics require similar caution: appropriately timed deposition may reinforce vascular and root tissues, whereas excessive lignification can restrict root elongation and resource acquisition. The relevant trait is consequently the amount, composition, timing, and anatomical location of deposition rather than total lignin alone.

Carotenoid regulation provides a complementary mechanism through photoprotection and abscisic acid metabolism. Heterologous expression of the foxtail millet transcription factor *SiLRL1* in *Arabidopsis thaliana* increased carotenoid and abscisic acid accumulation and improved drought tolerance [48]. This result identifies a promising cereal regulatory gene, but it does not yet demonstrate improved drought performance in foxtail millet itself. Functional validation in millet, preferably during reproductive development and under field drought, remains necessary. Rehydration represents an active metabolic phase rather than a simple reversal of drought. Water return restores hydraulic function and electron transport but can also produce transient oxidative stress. A winter wheat transcriptome–metabolome study associated rehydration timing with flavonoid metabolism, renewed growth, yield components, and specific agronomic traits [49]. Sampling only at maximum drought therefore cannot distinguish maintenance of function from damage tolerance or rapid repair. Photosynthetic recovery, electrolyte leakage, root regrowth, antioxidant resetting, reproductive continuation, and final yield should be treated as primary outcomes. For drought and rehydration, the most informative translational targets are therefore those associated with reproducible protection and recovery across relevant tissues, developmental stages, and agronomic outcomes. Intervention studies involving nutrients, priming compounds, or microorganisms should be separated from endogenous genotype responses because they may reduce stress exposure, modify signaling, or alter metabolism directly. Multi-environment validation is more informative than maximizing metabolite fold change under a single controlled treatment.

## 5. Salinity and Alkalinity: Integrating Osmotic, Ionic, and High pH Signals

Salinity combines osmotic stress with ion toxicity, whereas alkaline salts additionally impose high pH, carbonate chemistry, and nutrient availability constraints. These conditions should not be treated as interchangeable. Salt acclimation requires Na^+^ exclusion or sequestration, K^+^ retention, osmotic adjustment, membrane protection, and sustained photosynthesis; specialized metabolites interact with these processes but cannot replace ion transport systems. Rice studies demonstrate substantial cultivar and tissue specificity. Contrasting cultivars develop distinct physiological and metabolomics responses under salinity rather than a universal chemical signature [50]. In rice roots, integrated transcriptome and metabolome profiling associated flavonoid biosynthesis with the responses of both tolerant and sensitive cultivars, whereas galactose metabolism more clearly distinguished the tolerant cultivar [51]. These findings identify candidate pathways but remain associative until the proposed genes or metabolites are perturbed. Earlier rice metabolomics similarly identified serotonin and gentisic acid among metabolites that differentiated tolerant from sensitive cultivars, supporting their use as candidate markers rather than proven effectors [52].

Cross-cereal studies reinforce this context dependence. Salt-stressed maize shoots showed coordinated changes in mitogen-activated protein kinase and hormone signaling, transcription factor families, phenolic acids, flavonoids, benzoxazinoids, osmolytes, and membrane-related metabolites [53]. These findings should therefore be interpreted as associative evidence, consistent with the Evidence Assessment Framework in Section 3.5, until the highlighted metabolites or regulators are functionally validated. A synthetic allotetraploid wheat inherited flavonoid-associated salinity responses resembling those of its more tolerant parent, linking Polyploid inheritance with redox and metabolic adjustment [42]. In foxtail millet, contrasting genotypes under saline–alkaline stress differed in phenylpropanoid, flavonoid, Ca^2+^ signaling, and energy-related networks [54]. The contribution of flavonoids to salinity tolerance may also vary with their chemical form and intracellular distribution. Glycosylation, acylation, and transporter-mediated trafficking can alter flavonoid stability, retention, and distribution [28]. These features should therefore be considered when interpreting flavonoid-associated salinity responses, as outlined in Section 3.5.

## 6. Heat Stress and Thermal Recovery

Heat stress alters membrane organization, accelerates respiration, disrupts protein folding, and creates photochemical and oxidative imbalance. Short heat episodes primarily demand rapid proteostasis and membrane repair, whereas prolonged heat during flowering or grain filling impairs pollen viability, assimilate partitioning, yield, and grain quality. Secondary metabolite responses must therefore be interpreted within the heat shock factor–heat shock protein network rather than as an independent antioxidant pathway. Functional evidence from barley links the heat shock factor HvHsfA6a with thermotolerance. Constitutive overexpression improved photosynthesis and antioxidant capacity, induced heat shock proteins, altered secondary and central metabolism, and increased jasmonic acid accumulation [55]. The constitutive design nevertheless requires assessment for growth, phenology, and yield penalties under non-stress and field conditions. Flavonoid glycosylation provides stronger causal evidence in rice. *OsDUGT1* overexpression increased heat survival, whereas knockout reduced flavonoid abundance, reactive oxygen species control, and survival; biochemical assays confirmed broad flavonoid glycosyltransferase activity [37]. A second module showed that MYB61 directly activates *UGT706F1*, which glycosylates flavonoids and improves rice heat tolerance; knockout and overexpression lines supported necessity and sufficiency more strongly than correlative metabolomics alone [56]. Together, these studies highlight the importance of considering flavonoid chemical form, rather than total flavonoid abundance alone, when interpreting heat tolerance. Glycosylation can alter metabolite stability, transport, sequestration, and the balance between aglycones and conjugates, as outlined in Section 3.5.

Heat-induced increases in phenolic or anthocyanins do not necessarily improve grain value. Concentration per unit dry mass can rise when starch deposition and grain mass decline, creating an apparent enrichment caused by reduced dilution. To distinguish growth dilution from genuine biosynthetic increases, absolute metabolite content per grain or organ should be measured alongside concentration per unit dry weight. Nutritional interpretation should additionally consider bioavailability, extractability, processing stability, and final yield. Thermal recovery and priming should be incorporated into experimental designs. Recovery establishes whether membrane integrity, photosynthesis, reproductive growth, and metabolic homeostasis resume after heat is removed.

## 7. Waterlogging, Hypoxia, and Reoxygenation

Waterlogging restricts oxygen diffusion into the root zone, suppresses mitochondrial oxidative phosphorylation, and reduces adenosine triphosphate production. Plants consequently increase glycolysis and fermentation, in which pyruvate decarboxylase and alcohol dehydrogenase regenerate NAD^+^ and sustain limited substrate-level energy production [57]. Ethylene signaling and group VII ethylene response factors regulate oxygen sensing, carbohydrate use, and hypoxia-responsive transcription. Specialized metabolism contributes to waterlogging tolerance by interacting with redox regulation, carbohydrate conservation, and cell wall organization. In barley, roots displayed stronger transcriptional responses than leaves, while the tolerant genotype activated stress-responsive genes more rapidly than the sensitive genotype [39]. The tolerant genotype also maintained greater sugar availability, accumulated less lactate, and showed stronger pyruvate decarboxylase and alcohol dehydrogenase responses. Phenylpropanoid-pathway genes, including cinnamoyl coenzyme A reductases and peroxidases, were associated with increased cell wall biogenesis and redox control [39]. These results show that waterlogging tolerance does not depend simply on greater fermentation. Successful acclimation requires sufficient energy production, slower carbohydrate depletion, control of cytosolic acidification, and maintenance of root structure. The phenylpropanoid responses in this study should therefore be interpreted according to the Evidence Assessment Framework in Section 3.5, with the available transcript–metabolite relationships supporting associative rather than causal inference.

Reoxygenation is biologically distinct from the preceding hypoxic phase. Restoration of oxygen and light rapidly reactivates mitochondrial and chloroplast electron transport, which can generate reactive oxygen species, lipid peroxidation, and membrane injury [57]. Antioxidant systems, jasmonate signaling, redox regulation, and membrane repair therefore influence post-flood recovery rather than hypoxia survival alone. Future cereal experiments should include sampling during waterlogging, early drainage, and later recovery. Root viability, hydraulic conductance, electrolyte leakage, reactive oxygen species, antioxidant redox couples, phenylpropanoid turnover, regrowth, and final yield should be measured. Without recovery data, sustained metabolic activity during waterlogging cannot be distinguished from delayed cellular injury.

## 8. Potentially Toxic Elements: Chelation, Immobilization, and Redox Control

Cadmium, arsenic, and related potentially toxic elements disrupt electron transport, nutrient homeostasis, membrane integrity, enzyme activity, and cellular redox balance [58]. Their accumulation in edible cereal tissues also creates a direct food safety risk. Roots provide the first major barrier; tolerance and restricted grain loading therefore depend on uptake selectivity, cell wall binding, chelation, vacuolar sequestration, xylem loading, node distribution, and phloem transport [58]. Secondary metabolites contribute mainly through redox regulation, metal interaction, and structural modification, but they function alongside glutathione, phytochelatins, organic acids, and membrane transporters. In sorghum roots, cadmium exposure altered chalcone synthase, chalcone isomerase, flavonoid hydroxylase, and methyltransferase genes and changed the abundance of naringenin, apigenin, luteolin, taxifolin, and related flavonoids [59]. The same study identified enrichment of flavonoid, phenylpropanoid, glutathione, and ATP-binding cassette transporter pathways. These coordinated changes identify flavonoid metabolism as a candidate component of the cadmium response. According to the Evidence Assessment Framework in Section 3.5, however, the available associations do not by themselves establish direct metal chelation or causal detoxification.

Rice provides stronger evidence for lignin-mediated structural defense. Cadmium activated glutathione and phenylpropanoid pathways, increased root lignin content, thickened cell walls, and altered the expression of cadmium transporters [60]. Comparisons among rice materials with contrasting lignin contents further associated increased lignification with lower grain cadmium accumulation and improved yield under cadmium exposure. Lignification may immobilize cadmium within root apoplastic barriers and restrict long-distance transport, but excessive deposition can impair root elongation and nutrient acquisition. The relevant phenotype is therefore appropriately localized lignification rather than a general increase in total lignin.

Arsenic must be evaluated according to chemical species. In flooded rice soils, arsenite enters roots predominantly through the silicon influx transporter Lsi1, while Lsi2 promotes arsenite efflux towards the xylem; disruption of these transporters reduces arsenic accumulation in shoots and grain [61]. Arsenic sequestration also depends on phytochelatin complexes and ATP-binding cassette transporters. OsABCC1 restricts arsenic transfer to rice grain by sequestering arsenic complexes in vacuoles of phloem companion cells within the nodes [62]. Interpretation of chelation or immobilization should therefore consider the chemical and spatial evidence available, including elemental speciation, binding analyses, imaging, subcellular fractionation, cell wall composition, anatomy, transporter expression, and root-to-grain translocation, as appropriate to the proposed mechanism. The breeding target is not maximal secondary metabolite accumulation. It is a coordinated phenotype that restricts toxic-element entry into edible tissues while maintaining root function, mineral nutrition, biomass, grain yield, and nutritional quality.

## 9. Combined Stresses and Non-Additive Metabolic Regulation

Field-grown cereals commonly experience simultaneous or sequential stresses, and their combined effects cannot be predicted by simply adding the responses to each stress. Shared signals, including reactive oxygen species, Ca^2+^, abscisic acid, jasmonate, and mitogen-activated protein kinase pathways, may reinforce one another or produce antagonistic responses [63]. Stress order, intensity, duration, tissue, and developmental stage further determine whether previous exposure induces acclimation, priming, metabolic saturation, or vulnerability. Drought and heat illustrate this non-additivity. Drought promotes stomatal closure to conserve water, whereas heat dissipation often benefits from continued transpiration. Combined exposure therefore creates a physiological conflict between water conservation and leaf cooling. At the metabolic level, competition for carbon skeletons, reducing equivalents, and activated sugars may constrain both growth and specialized metabolite synthesis. Conversely, reduced biomass can increase metabolite concentration through a dilution effect without increasing pathway flux.

A pathway activated by the first stress may respond weakly to a subsequent stress because it is already induced, substrate limited, transcriptionally repressed, or undergoing recovery. Combined-stress experiments should therefore include an unstressed control, each stress, the combined treatment, and a recovery phase. Without this factorial structure, a genuinely non-additive response cannot be separated from the effect of the dominant individual stress. Wheat exposed to combined cadmium and salinity provides a clear cereal example. The combined treatment caused greater growth inhibition, chlorophyll loss, superoxide accumulation, and lipid peroxidation than either stress alone [64]. Cadmium reduced salinity-induced *SOS1* expression and increased Na^+^ accumulation, whereas salinity suppressed several cadmium-uptake transporters and reduced Cd^2+^ accumulation. The combination therefore intensified overall injury while producing antagonistic effects on specific ion-transport processes. A complementary example is provided by summer maize exposed to combined drought and heat stress. Yuan et al. [65] used non-targeted rhizosphere metabolomics together with microbial community profiling to compare drought, heat, and combined drought–heat stress. After 15 days, combined drought and heat reduced aboveground biomass by 51.7% and root biomass by 34.5% relative to the control. The combined treatment also produced distinct rhizosphere metabolic responses, with differential abundance of phenol ethers, fatty acyls, organooxygen compounds, allyl-type 1,3-dipolar organic molecules, and organic nitro compounds. Pathway analysis further revealed different patterns under combined stress compared with the individual stresses, including increased representation of ABC transporters and valine, leucine, and isoleucine biosynthesis pathways. The authors further proposed that combined drought and heat stimulated root secretion of L-valine, which was associated with recruitment of Gemmatimonadota and improved nutrient acquisition. These findings demonstrate that combined drought and heat can reorganize rhizosphere metabolism and plant–microbe interactions in ways that are not readily predictable from single-stress responses alone [65]. Interpretation of combined-stress responses should therefore recognize that the direction, magnitude, and functional significance of a metabolic response may change when stresses occur simultaneously or sequentially. Single-stress metabolomic signatures should not automatically be extrapolated to combined-stress conditions. Accordingly, interpretation should follow the Evidence Assessment Framework in Section 3.5, with emphasis on modules that show consistent links among molecular regulation, metabolic responses, physiological protection, and agronomic performance rather than simply increased metabolite abundance. Candidate modules must be tested across genotypes, developmental stages, stress sequences, and field environments and evaluated for recovery, yield, grain quality, and food or feed safety. Across drought, salinity and alkalinity, heat, waterlogging, potentially toxic elements, and combined stresses, specialized metabolites converge on redox control, membrane and photosystem protection, ion homeostasis, cell-wall reinforcement, carbon allocation, and recovery, as synthesized in Figure 3. Representative cereal studies, experimental contexts, molecular components, metabolite classes, physiological outcomes, and levels of mechanistic support are compared in Table 2.

## 10. Cellular Integrity as the Unifying Physiological Endpoint

“Maintenance of cellular integrity” should refer to measurable preservation of plasma membrane permeability, organelle structure, thylakoid function, mitochondrial respiration, vacuolar sequestration, and cell wall continuity. Reactive oxygen species (ROS) are generated and perceived within specific cellular compartments; consequently, bulk tissue ROS measurements cannot identify where injury originates or where protection occurs [69]. Secondary metabolites contribute differently according to their chemical form and localization. Carotenoids function within plastids and photosynthetic complexes, whereas flavonoids may accumulate near chloroplasts, nuclei, membranes, or in vacuoles [11,27]. Lignin and wall-bound hydroxycinnamates modify cell wall permeability and mechanical strength, while glycosylated metabolites are commonly stored in vascular pools. The subcellular organization of specialized-metabolite biosynthesis, modification, transport, storage, and structural deployment is illustrated in Figure 4. Antioxidant capacity is frequently overinterpreted. Extract-based assays using 2,2-diphenyl-1-picrylhydrazyl, 2,2′-azinobis (3-ethylbenzothiazoline-6-sulfonic acid), or ferric reducing antioxidant power quantify chemical reactivity under defined in vitro conditions; such measurements should therefore be interpreted as in vitro biochemical evidence rather than direct evidence of physiological ROS scavenging in cereal cells, as outlined in Section 3.5 [70]. In vivo effectiveness depends on reaction kinetics, concentration, regeneration, molecular targets, and proximity to the site of ROS production. Flavonoids may also regulate enzymes, ion transport, and signaling rather than act solely through direct radical scavenging [11]. Mechanistic studies should therefore combine metabolite measurements with H_2_O_2_ and superoxide localization, lipid peroxidation, electrolyte leakage, glutathione and ascorbate redox states, organelle performance, and genetic perturbation.

Chemical tailoring provides another determinant of function. Glycosylation alters metabolite solubility, reactivity, cellular localization, and bioactivity, while acylation and methylation can modify stability, retention, and membrane association [71]. Glutathione S-transferase ligandins, multidrug and toxic compound extrusion transporters, ATP-binding cassette transporters, and vesicular trafficking contribute to flavonoid movement and vascular sequestration [28]. Total flavonoid assays and untargeted feature counts obscure differences among aglycones, glycosides, acylated derivatives, and wall-bound forms. Accordingly, interpretation of individual metabolites should consider chemical identity, annotation confidence, and the evidence supporting their physiological function, consistent with the framework in Section 3.5. Cellular integrity is ultimately a systems property. Membrane preservation supports ion transport and organelle function, stable thylakoids sustain carbon and reducing-power supply, controlled vascular sequestration prevents reactive intermediates from damaging cytosolic targets, and appropriate wall reinforcement restricts uncontrolled ion movement without preventing growth. The adaptive value of specialized metabolism therefore depends on coordinated biosynthesis, modification, transport, localization, and physiological recovery rather than on the concentration of a single antioxidant class [7].

## 11. Multi-Omics, Metabolic Flux, and Causal Inference

Metabolomics platforms support different levels of inference. Targeted methods quantify predefined compounds, usually with authentic standards, and provide greater chemical and quantitative confidence. Untargeted high-resolution mass spectrometry captures broader chemical diversity but generates many unresolved or putatively annotated features [13]. Widely targeted liquid chromatography–tandem mass spectrometry occupies an intermediate position by combining large spectral libraries with multiple reaction monitoring, although the resulting values are generally relative rather than absolute concentrations [72]. Cross-study comparisons must therefore distinguish absolute concentrations, relative signal intensities, and unidentified molecular features. Analytical quality must be secured before omics integration. Studies should report biological replication, sample randomization, extraction and injection order, pooled quality control samples, blanks, internal standards, signal drift correction, missing value treatment, and multiple testing control [13]. Technical injections assess analytical precision but do not increase biological replication. Metabolite names should also reflect identification confidence, ranging from authentic standard confirmation to tentative class or molecular formula assignment [19]. Raw spectra, processed peak tables, metadata, and analysis code should be deposited in accessible repositories to support reanalysis and cross-study comparison.

Integrated transcriptome–metabolome studies commonly use correlation networks, weighted gene coexpression network analysis, and pathway enrichment to identify coordinated modules. These approaches are valuable for identifying coordinated candidate modules, but their biological interpretation should follow the Evidence Assessment Framework in Section 3.5. In particular, network associations should not be interpreted as causal mechanisms without appropriate experimental validation. Complementary approaches, including temporal analysis, genetic perturbation, biochemical assays, metabolic flux analysis, spatial localization, complementation, and chemical rescue, can strengthen causal interpretation when they directly test the proposed mechanism [18]. In rice, *OsCHI3* knockout and overexpression connected flavonoid and abscisic acid metabolism with drought tolerance [46]. Similarly, *OsDUGT1* perturbation and enzyme assays linked flavonoid glycosylation with heat tolerance [37].

Machine learning can prioritize candidate metabolites and genes, but prediction does not establish biochemical causality. Random sample splitting may leak genotype, environment, tissue, or analytical batch information between training and testing sets. Feature selection conducted before cross-validation can also inflate apparent accuracy [73]. Grouped or nested cross-validation, locked preprocessing, permutation testing, uncertainty estimates, and independent validation are therefore essential [74]. Models should predict meaningful outcomes, such as recovery, yield, or grain quality, rather than merely reproduce treatment labels. Current machine learning models in cereal stress research are predominantly based on seedling-stage or controlled laboratory data, and their predictive power for field conditions and reproductive-stage traits remains limited. Therefore, model performance should be independently evaluated across developmental stages, genotypes, environments, and field conditions before ML-derived predictors are considered broadly transferable. For secondary-metabolite studies, the value of multi-omics integration therefore depends on whether independent molecular, biochemical, physiological, and agronomic evidence converges on the proposed mechanism, rather than on the number of omics platforms combined.

## 12. Translational Value for Breeding and Metabolic Engineering

Breeding requires metabolite markers that are heritable, reproducible, measurable at an operational stage, and consistently associated with agronomic performance. In maize, metabolite-based genome-wide association analysis linked kernel metabolic variation with numerous genetic loci and identified metabolite features associated with kernel weight [75]. Comparative metabolic and phenotypic association studies in rice and maize further demonstrated that genetic control can differ among tissues and species, although conserved loci can reveal candidate metabolic genes [76]. Metabolite markers must therefore be validated across developmental stages, populations, and environments before routine selection. Metabolites measured only after severe injury may distinguish tolerant and sensitive genotypes but remain impractical for breeding. More useful traits may include constitutive precursor pools, inducible metabolite ratios, recovery kinetics, or alleles controlling pathway regulation, tailoring, transport, and sequestration. Population structure, environmental variation, tissue composition, and analytical batch must be controlled when metabolite quantitative trait loci or genomic prediction models are developed.

Genome editing can target transcription factors, biosynthetic enzymes, tailoring enzymes, transporters, or cis-regulatory elements. Editing an upstream regulator may coordinate several pathway branches but increases pleiotropic risk; modifying a late enzyme or transporter can produce a more specific chemical phenotype. Promoter editing generated quantitative variation in maize yield-related traits without relying exclusively on complete gene knockout [77]. In rice, deletion of a small regulatory region within the *IPA1* promoter reduced the trade-off between panicle size and tiller number and increased grain yield [78]. These studies support cis-regulatory editing as a strategy for tuning expression, although stress-responsive metabolic genes require validation in their native tissue and environmental context. Validated secondary metabolite modules provide stronger targets than unstructured lists of stress-responsive compounds. *OsCHI3* connects flavonoid biosynthesis and abscisic acid regulation with rice drought tolerance, while *OsDUGT1* links flavonoid glycosylation with heat survival. These examples demonstrate that pathway entry and chemical tailoring can both alter stress performance. Nevertheless, seedling survival alone does not justify deployment. Each edited module must be evaluated for root growth, phenology, reproductive development, yield, grain composition, and unintended metabolic effects.

Agronomic treatments can also change specialized metabolism, but their mechanisms should be separated into reduced stress exposure, signaling priming, and direct metabolic modification. A treatment that improves water or nutrient status may normalize metabolite profiles indirectly; this response should not automatically be described as activation of a protective pathway. Field evaluation should include economic feasibility, treatment persistence, environmental effects, and interactions with genotype. Translation must preserve both productivity and product quality. Increased anthocyanin, phenolic acid, or carotenoid concentration is valuable only when absolute content, bioavailability, processing quality, and grain yield are maintained. Potentially toxic element studies must additionally demonstrate lower contaminant accumulation in edible tissues, while cyanogenic pathways require explicit food and feed safety assessment. The translational value of a candidate identified through the Evidence Assessment Framework in Section 3.5 should ultimately be evaluated across the relevant stress, developmental stage, regulator or allele, metabolite pathway and chemical form, tissue and subcellular compartment, physiological function, and agronomic outcome. Multi-environment validation is therefore not merely the final deployment step; it determines whether a molecular mechanism has agricultural value. The progression from analytical discovery and candidate prioritization to causal validation, metabolic design, breeding, and field deployment is summarized in Figure 5.

## 13. Crop-Specific Synthesis, Trade-Offs, and Interpretive Framework

### 13.1. Crop-Specific Mechanistic Patterns

Rice provides the strongest direct genetic evidence linking specialized metabolism with cereal stress tolerance, but the mechanisms remain tissue- and stress-specific. *OsCHI3* overexpression increased flavonoid accumulation and drought tolerance, whereas CRISPR–Cas9 knockout increased drought sensitivity; exogenous abscisic acid partially rescued the mutant phenotype [46]. Under heat stress, *OsDUGT1* overexpression improved rice survival, while knockout reduced flavonoid glycosides, antioxidant capacity, and thermotolerance [37]. These modules operate through different enzymes, chemical forms, and stress contexts and therefore do not support a universal rice metabolite ideotype. Rice improvement should instead distinguish root ion exclusion, leaf photoprotection, recovery, reproductive-stage tolerance, and grain quality targets.

Wheat evidence is more strongly distributed across developmental stages and recovery phases. Integrated transcriptomic and metabolomics analysis showed that flavonoid metabolism was reorganized during rehydration and associated with resumed winter-wheat growth, demonstrating that recovery is not simply the reversal of drought responses [49]. Wheat improvement should therefore distinguish establishment-stage root resilience, canopy protection during terminal drought or heat, reproductive tolerance, and maintenance of grain quality. Correlative multi-omics candidates require validation in genetically diverse wheat backgrounds before they can be treated as breeding targets.

Maize has extensive transcriptome–metabolome and genetic-association resources but comparatively fewer direct perturbation studies of specialized-metabolite pathways. Salt-treated maize shoots showed coordinated changes in phenolic acids, flavonoids, benzoxazinoid, hormones, and ion homeostasis, although the evidence was explicitly correlative [53]. Metabolome-wide association analysis has also connected kernel metabolites with genetic loci and agronomic traits, illustrating the value of maize diversity panels for candidate discovery [75]. Future studies should test candidate enzymes and transporters across hybrid backgrounds and determine whether biochemical protection persists during reproductive stress and grain filling.

Barley provides a useful bridge between vegetative stress physiology and grain or feed quality. Under waterlogging, the tolerant genotype activated root responses earlier, conserved sugars, accumulated less lactate, and showed stronger fermentation and wall-associated phenylpropanoid responses than the sensitive genotype [39]. Direct overexpression of *HvHsfA6a* also improved heat tolerance through changes in photosynthesis, antioxidant systems, heat-shock responses, and primary and specialized metabolism [55]. These studies support regulator–metabolite modules but also show that tolerance depends on energy conservation, structural protection, and recovery rather than metabolite accumulation alone.

Sorghum and millets contain distinctive chemistry that creates both opportunities and safety constraints. Cadmium exposure in sorghum roots produced coordinated flavonoid-pathway changes, but the transcript–metabolite associations did not establish direct chelation or causal detoxification [59]. Sorghum dhurrin contributes to defence and nitrogen metabolism, yet its hydrolysis can release hydrogen cyanide; stress-related pathway manipulation must therefore include cyanogenic-potential and forage-safety measurements [79]. In foxtail millet, *SiLRL1* increased carotenoids, abscisic acid, and drought tolerance when expressed in *Arabidopsis thaliana*, but its function has not yet been validated through genetic perturbation in millet itself [48].

### 13.2. Crop-Specific Research Gaps

Recent functional studies in maize and sorghum demonstrate that the evidence base is beginning to extend beyond omics-level association, although important gaps remain. In maize, functional characterization of *Zm4CL8* using virus-induced gene silencing showed that disruption of this phenylpropanoid-associated gene increased sensitivity to drought and salinity, providing experimental support for its role in abiotic stress adaptation [80]. CRISPR–Cas9 editing of *ZmPMT1* further demonstrated that targeted manipulation of a lignin-biosynthetic gene can alter phenolic metabolism and lignin structure, illustrating the feasibility of experimentally resolving specialized-metabolite pathway function in maize [81]. In sorghum, genetic analysis of *SbYR1* linked a defined mutation with altered flavonoid, jasmonate, and abscisic acid metabolism and stress-related growth responses, while functional analysis of *Bmr12*, which encodes caffeic acid O-methyltransferase, demonstrated that altered *Bmr12* function can modify flavonoid-associated defense against fall armyworm [82,83]. These examples strengthen cross-crop comparisons by showing that functional genetic evidence can connect specialized-metabolite pathways with physiological or defense phenotypes beyond correlative metabolomics. By contrast, evidence remains substantially weaker for oat, rye, and triticale. For these crops, available studies are dominated by descriptive metabolomic or biochemical profiling, with limited evidence directly linking candidate specialized-metabolite genes or pathways to stress phenotypes through genetic perturbation. Thus, for oat, rye, and triticale, functional validation remains a major research priority. Future work should combine genome-wide association or metabolomic discovery with gene editing, allele contrasts, biochemical validation, spatial analysis, and physiological or agronomic rescue experiments to establish whether candidate metabolite pathways are causal rather than merely stress-associated.

Across cereals, conserved regulator and enzyme families do not necessarily produce identical chemical or physiological outcomes. Gene copy number, substrate specificity, tissue expression, chemical tailoring, and transport can differ among crops. Comparative functional genomics should therefore test orthologous modules in their native cereal backgrounds rather than infer conserved function solely from sequence similarity or heterologous expression [7].

### 13.3. Growth–Defense Trade-Offs and Metabolite-Class Interpretation

The relationship between specialized-metabolite accumulation and tolerance is frequently nonlinear. Moderate pathway activation may protect membranes, photosystems, or cell walls, whereas constitutive or excessive activation can divert carbon, nitrogen, reducing power, and activated sugars from growth and reproduction. Negative or context-dependent responses provide important evidence against interpreting metabolite accumulation as universally beneficial. For example, the sorghum *sbyr1* mutant exhibited enhanced stress resistance accompanied by elevated flavonoid-, jasmonate-, and abscisic acid-related responses, but also showed stunted growth, illustrating a potential trade-off between defense activation and plant productivity [82]. Similarly, drought in maize can modify root suberization and lignification together with aquaporin activity and hydraulic conductivity. Although increased structural barriers may contribute to drought acclimation, excessive or tissue-specific changes can restrict water transport and therefore do not necessarily indicate improved hydraulic performance [84]. These examples demonstrate that a stronger defense or structural response may improve one component of stress adaptation while imposing costs on growth, resource allocation, or water transport. Experiments should use graded stress intensities and recovery treatments to identify induction, saturation, and injury thresholds. Metabolite concentration should also be reported together with tissue mass and total content because growth inhibition can increase concentration per unit dry mass without increasing synthesis per plant.

Recovery should be considered an essential component of drought-response experiments rather than an optional endpoint. Many published drought studies focus primarily on peak-stress measurements and do not adequately characterize the rehydration phase, which limits interpretation of whether stress-induced metabolic changes represent persistent adaptation, transient acclimation, or injury. Rehydration can substantially alter metabolite profiles and physiological responses, and recovery trajectories may differ among metabolites, tissues, genotypes, and developmental stages. Therefore, drought experiments should, where feasible, include sampling at peak stress and during rehydration to distinguish stress resistance from recovery capacity and to identify persistent metabolic signatures or stress-memory responses.

Interpretation must reflect metabolite chemistry. Phenolic acids should be separated into soluble, conjugated, and wall-bound fractions because these pools have different redox and structural functions. Flavonoid studies should resolve aglycones, glycosides, and acylated forms rather than rely on total-flavonoid assays. Lignin should be evaluated through deposition site, polymer amount, monomer composition, anatomy, and hydraulic function rather than pathway enrichment alone. Carotenoid studies should distinguish total abundance, xanthophyll-cycle composition, photoprotection, and cleavage into abscisic acid precursors or other apocarotenoids [27].

Crop-specific defensive compounds require additional caution. Benzoxazinoids should be examined in relation to root exudation, microbial recruitment, and biotic interactions rather than automatically classified as antioxidants. Dhurrin studies should measure intact glycosides, turnover products, and cyanogenic potential. More generally, increased defense chemistry is agronomically valuable only when it preserves recovery, yield, grain quality, and food or feed safety. Taken together, these examples emphasize that the functional value of specialized-metabolite responses should be evaluated according to their net effects on stress protection, growth, hydraulic function, recovery, and final agronomic performance. A metabolite that increases strongly during stress may represent an adaptive response, a consequence of reduced growth and dilution, or a costly defense strategy. Accordingly, metabolite abundance alone should not be used as a proxy for stress tolerance without considering biomass accumulation, physiological function, recovery, and yield.

### 13.4. Interpretive Boundaries and Application of the Evidence Framework

Mechanistic interpretation should follow the Evidence Assessment Framework established in Section 3.5. Pathway enrichment and coordinated transcript–metabolite changes should be treated as associative evidence unless supported by additional experimental validation, whereas genetic perturbation, physiological rescue, and complementary biochemical or flux-based analyses can provide stronger causal support. In vitro biochemical assays should be interpreted as mechanistic evidence outside the intact plant and should not, by themselves, be equated with physiological function. Metabolite annotation confidence should likewise be considered when evaluating the strength of biological conclusions [13].

Evaluation of a proposed specialized metabolite mechanism should begin by determining whether stress exposure was measured and biologically relevant. The metabolite should be identified and quantified with adequate chemical confidence, and its tissue and subcellular localization should be compatible with the proposed function. Stronger mechanistic support requires causal perturbation of the relevant regulator, enzyme, transporter, or metabolite, followed by demonstration that the response improves recovery, yield, quality, or safety under an environmentally relevant condition. Claims that do not meet these requirements should be presented as associations or candidate mechanisms. A metabolite should be considered part of an adaptive mechanism only when its pathway is activated in the appropriate tissue and time window, its chemical form and localization support the proposed function, perturbation alters both chemistry and phenotype, and the benefit persists during recovery or realistic stress exposure. This framework converts variation among crops, genotypes, organs, and environments into biological information rather than treating heterogeneity as experimental noise.

## 14. Research Priorities and Future Directions

Future research should move beyond descriptive metabolite profiling and determine when specialized-metabolite reprogramming produces reproducible cellular protection and agronomic benefits.

**Improve experimental realism.** Stress exposure should be measured rather than assumed. Drought studies should report soil water potential, atmospheric demand, rooting volume, stress progression, and rehydration, while salinity studies should specify ion composition, electrical conductivity, osmotic potential, and pH [14]. Waterlogging experiments should quantify root-zone oxygen and include reoxygenation because hypoxia and post-drainage recovery represent distinct physiological phases [57]. Combined-stress designs should include an unstressed control, each individual stress, the complete combination, and recovery treatments [63].

**Resolve spatial, temporal, and flux dynamics.** Time-course sampling should distinguish early signaling, acclimation, injury, and recovery. Imaging mass spectrometry, laser-capture sampling, and subcellular fractionation should establish where candidate metabolites accumulate and whether their localization supports the proposed function [85]. Stable-isotope tracing and metabolic modelling should distinguish increased biosynthesis from altered degradation, transport, sequestration, or biomass dilution [86].

**Strengthen causal validation.** Correlation networks and pathway enrichment should be treated as hypothesis-generating evidence. Candidate regulators, enzymes, and transporters should be tested through allelic variation, knockout, overexpression, complementation, biochemical assays, localization, isotope tracing, and physiologically realistic chemical rescue [18]. A credible mechanism should connect molecular perturbation with chemical form, pathway flux, cellular protection, recovery, and whole-plant performance.

**Standardize chemical identification and data reporting.** Metabolite records should include molecular formulae, exact masses, retention times, diagnostic fragments, database identifiers, quantification methods, and annotation-confidence levels. Raw mass spectrometry data should be deposited in public repositories (e.g., MetaboLights) as a minimum data-sharing requirement, together with appropriate metadata and processed peak tables to facilitate independent verification, reanalysis, and metabolite reannotation. Metabolite annotation confidence levels (Levels 1–4) should be explicitly stated in Section 2 (Materials and Methods) so that the strength of metabolite identification is transparent and biological conclusions are not based on ambiguous annotations. Raw spectra, processed peak tables, metadata, and analytical scripts should also be deposited in accessible repositories to support reanalysis and reannotation [13,19]. Curated cereal spectral libraries and shared reference materials would further improve cross-study comparability.

**Evaluate crop-specific trade-offs and safety.** Candidate pathways should be tested in their native cereal backgrounds because gene duplication, substrate specificity, tissue expression, chemical tailoring, and transport can alter specialized-metabolite function among species [7]. Cyanogenic pathways require explicit safety evaluation because hydrolysis of cyanogenic glycosides can release hydrogen cyanide into food or forage [79]. Lignin- and metal-related targets should priorities controlled root retention with preserved growth, nutrient acquisition, yield, and grain safety rather than maximal lignification or metal sequestration [60].

**Prioritize field translation.** Promising regulator–enzyme–metabolite–transport modules should be tested across genotypes, developmental stages, environments, and management systems. Field trials should quantify stress exposure, recovery, phenology, resource-use efficiency, yield stability, grain composition, food or feed safety, and economic feasibility. Controlled-environment survival alone is insufficient because stress-adaptation mechanisms are often environment-specific and may not confer yield stability under field conditions [16].

Together, these priorities provide a practical route from pathway association to validated and deployable cereal-improvement targets. The minimum experimental, analytical, and translational evidence required to progress from pathway association to a validated cereal improvement target is summarized in Table 3.

## 15. Conclusions

Specialized metabolism is an integral component of cereal adaptation to abiotic stress. Through redox regulation, photosynthetic protection, membrane stabilization, cell wall reinforcement, ion and metal restriction, chemical sequestration, signaling, and recovery after stress, specialized metabolites connect molecular stress perception with cellular integrity and plant performance. Nevertheless, increased metabolite abundance or pathway enrichment alone does not demonstrate an adaptive mechanism. This review establishes a context-dependent framework in which stress sensing, kinase and hormonal signaling, transcriptional regulation, biosynthesis, chemical tailoring, transport, and compartmentation operate as coordinated modules. The function of each module depends on crop, genotype, tissue, developmental stage, stress intensity and sequence, resource availability, and recovery capacity. Chemical identity, localization, and turnover are therefore more informative than total metabolite concentration alone. The principal limitation of current cereal research is the gap between descriptive multiomic associations and direct functional evidence. Mechanistic confidence requires accurate chemical annotation, temporal and spatial resolution, metabolic flux analysis, genetic or biochemical perturbation, and validation at the cellular, whole plant, and agronomic levels. Evidence should progress from pathway association to causal validation and, ultimately, to performance across multiple environments. The most promising targets for breeding and metabolic engineering are not universally abundant metabolites, but context-defined modules that integrate regulators, enzymes, metabolites, transport processes, and physiological outcomes. These modules should be adjusted without imposing unacceptable penalties on growth, phenology, yield, grain quality, or food and feed safety. Applying this framework will move cereal specialized metabolite research from descriptive stress signatures towards reproducible and deployable strategies for climate-resilient crop improvement.

## Figures and Tables

**Figure 1:**
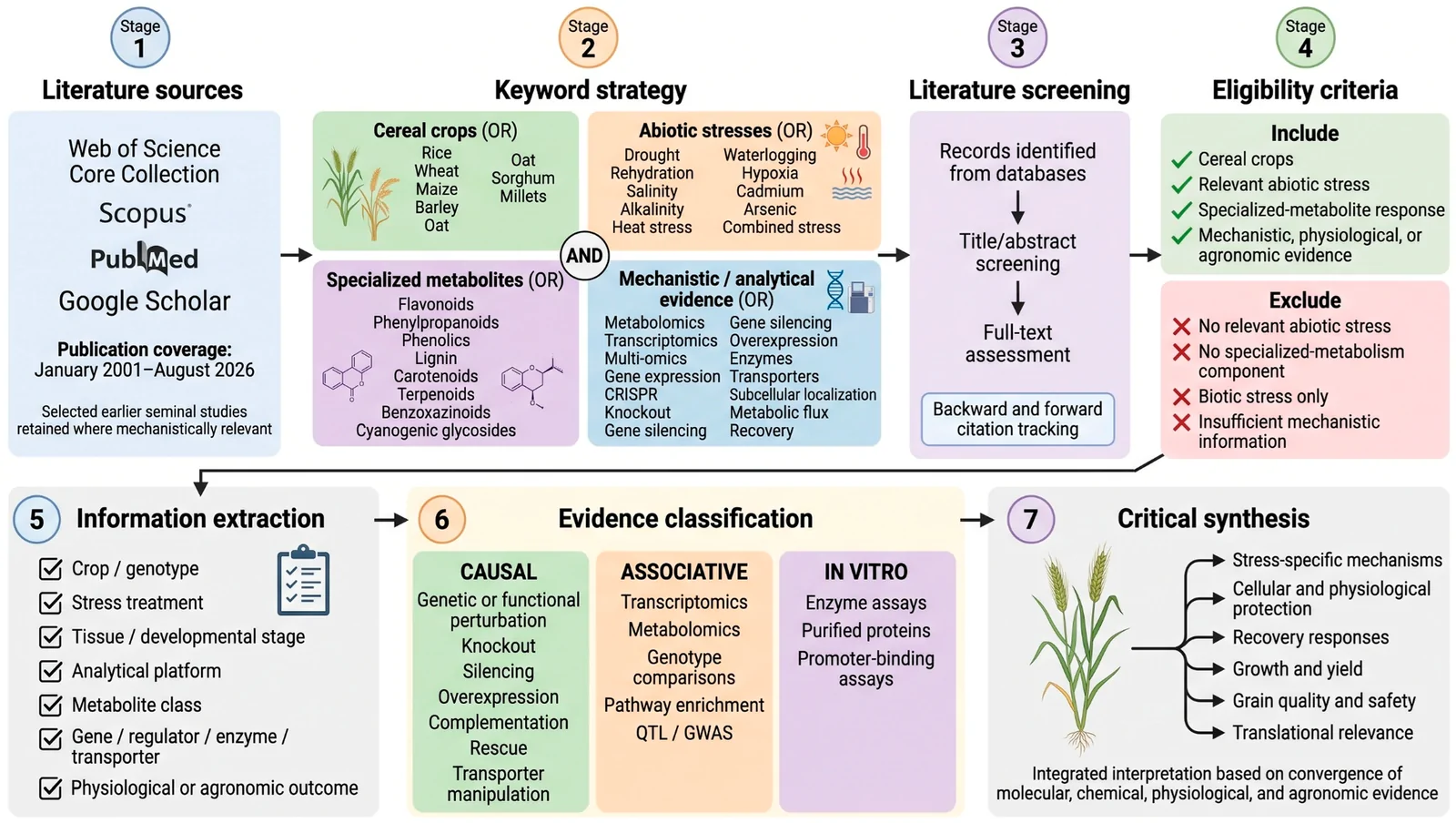
Structured literature-search and evidence-synthesis workflow used in this review. The workflow summarizes literature identification, keyword-based searching, screening and eligibility assessment, information extraction, classification of mechanistic evidence, and critical synthesis across molecular, chemical, physiological, and agronomic outcomes.

**Figure 2:**
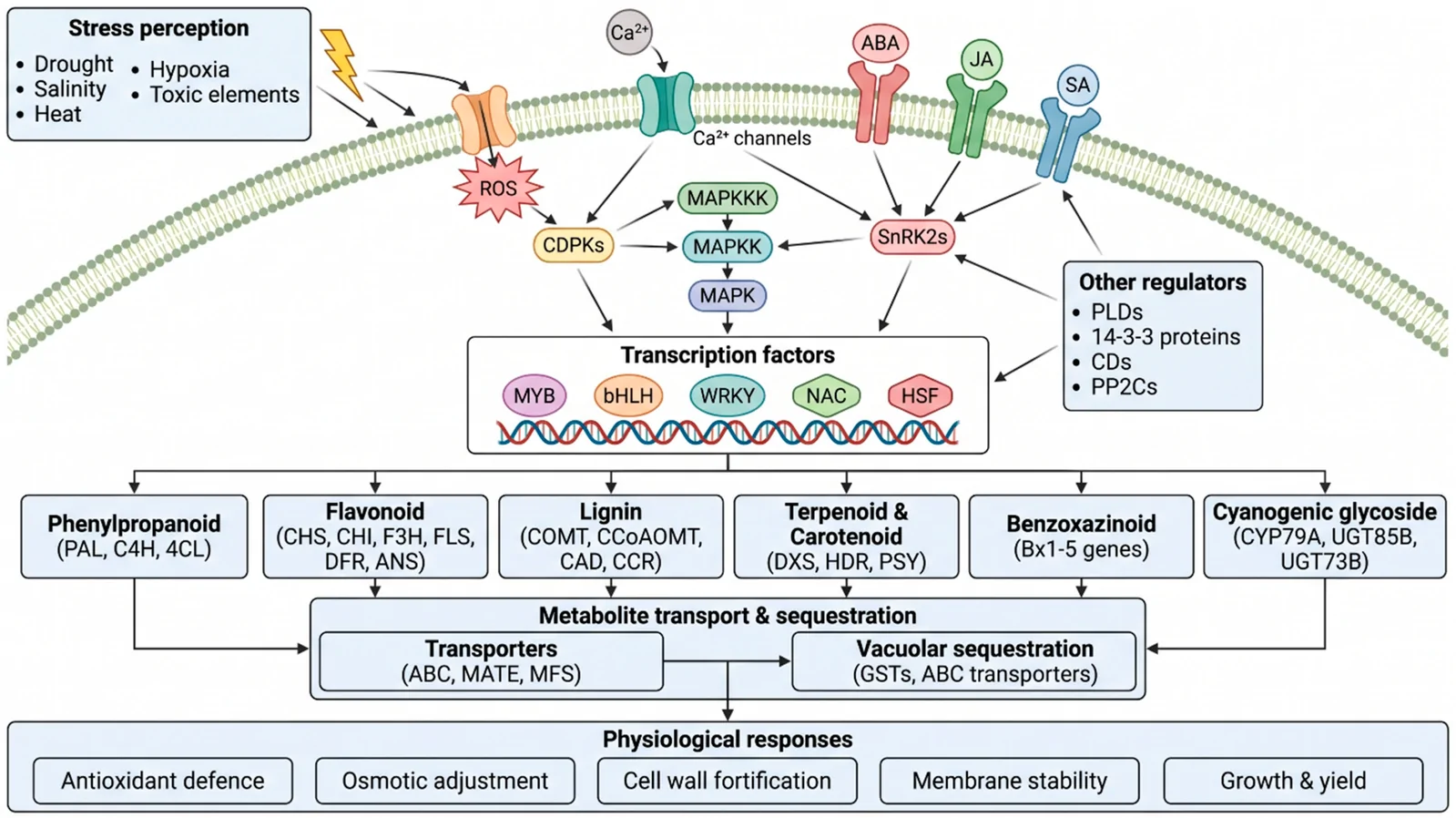
Integrated regulatory framework linking abiotic stress signaling with specialized-metabolite reprogramming in cereal crops. Abiotic stresses activate ROS, Ca^2+^, kinase, and hormone signaling networks that converge on transcriptional regulators and specialized-metabolite pathways. Metabolite modification, transport, and sequestration influence chemical form and cellular localization, thereby affecting stress protection, growth, and yield. Solid arrows indicate established regulatory relationships [5,20].

**Figure 3:**
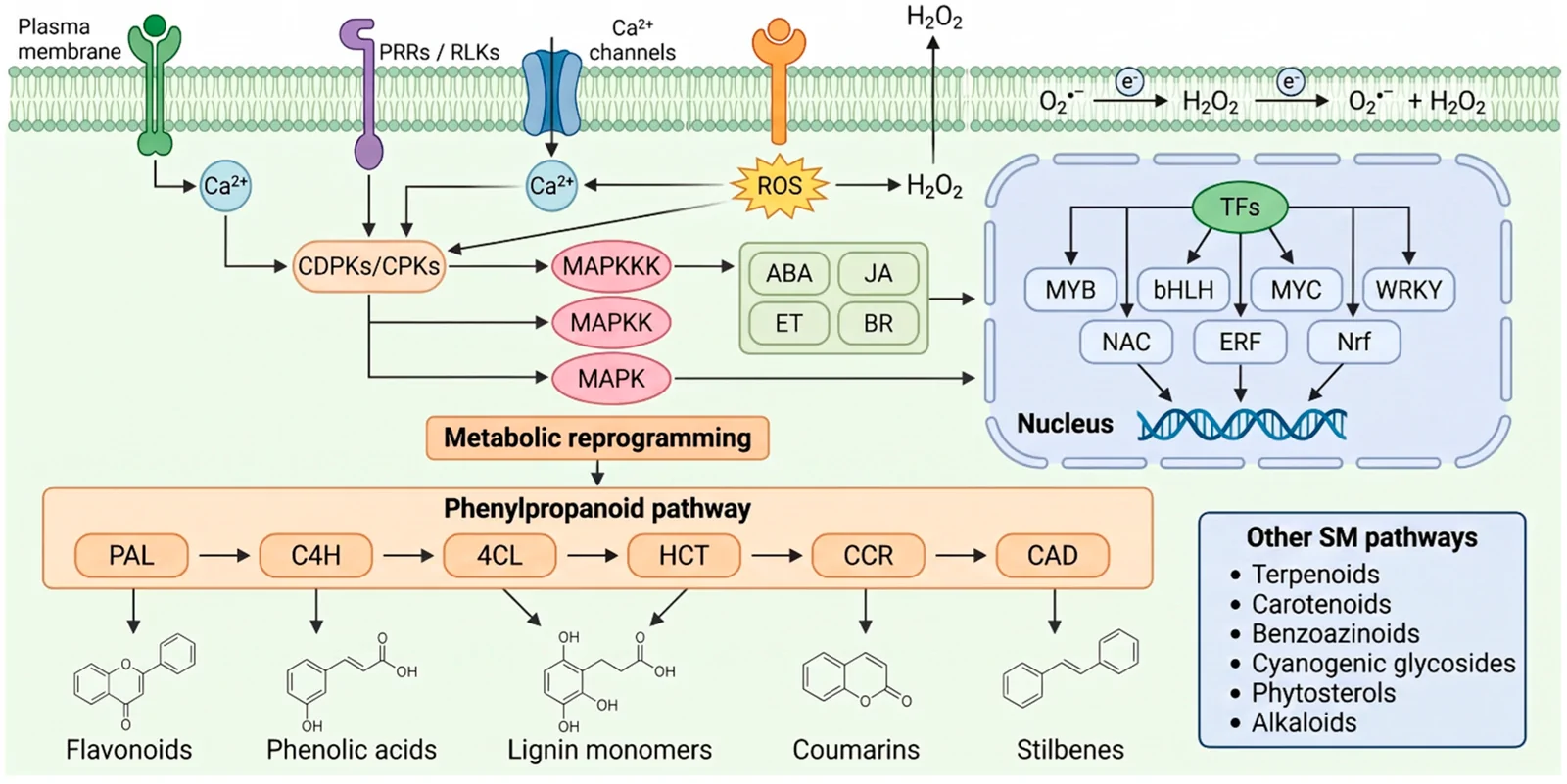
Integration of specialized-metabolite functions with whole-plant responses to abiotic stress in cereals. Abiotic stresses modify specialized-metabolite pathways and their interactions with antioxidant systems, ion homeostasis, membrane stability, cell wall organization, photosynthesis, and carbon allocation. The resulting responses vary with crop, genotype, tissue, developmental stage, stress intensity, duration, and sequence.

**Figure 4:**
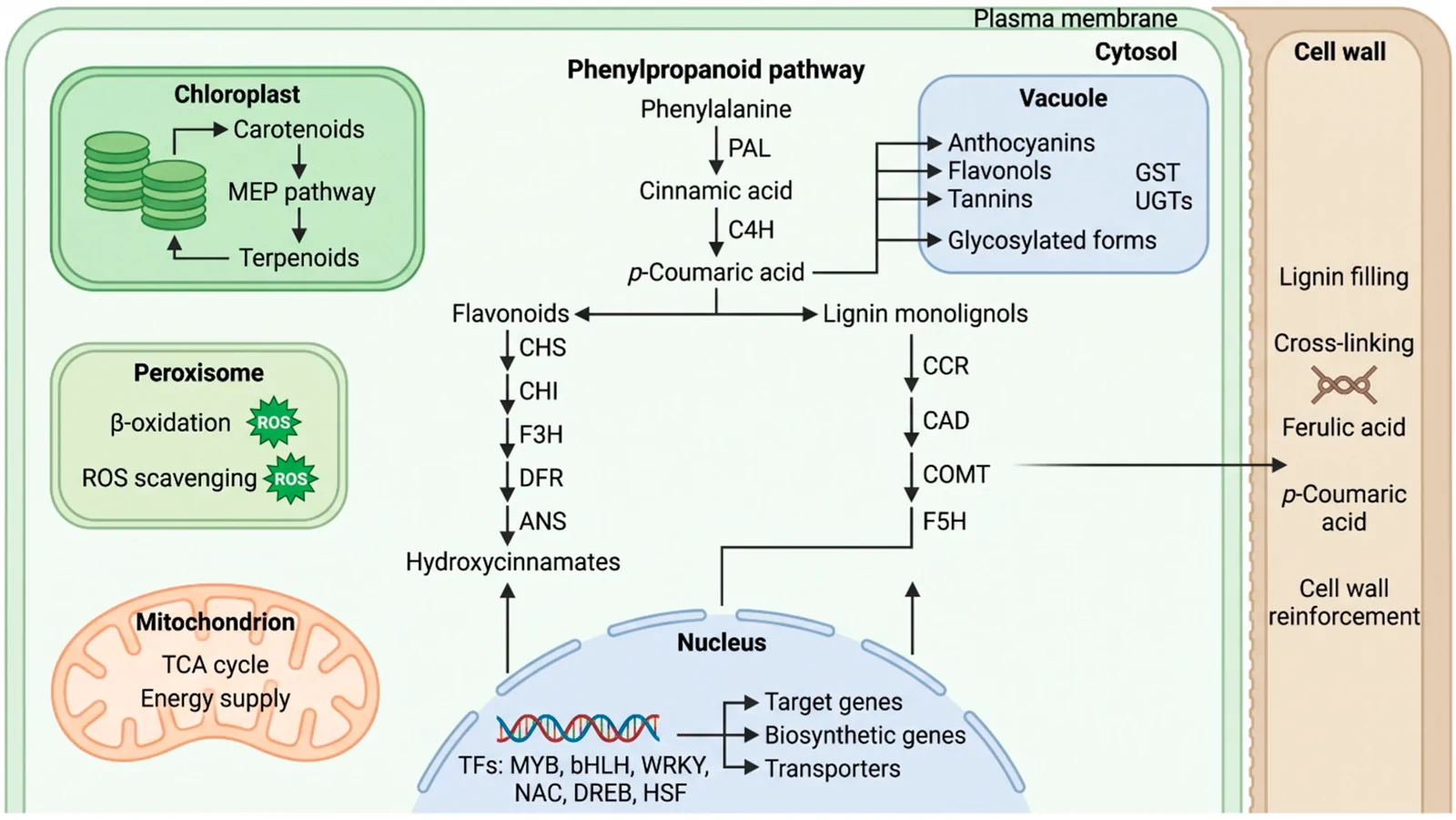
Subcellular organization of specialized-metabolite biosynthesis, modification, storage, and structural deployment in a cereal cell. Nuclear transcription factors regulate biosynthetic and transporter genes, while cytosolic, membrane-associated, plastidial, mitochondrial, and peroxisomal processes contribute to specialized-metabolite production and regulation. Chemical modification and transport determine metabolite accumulation, localization, and deployment in redox regulation, sequestration, photosynthetic protection, and cell wall reinforcement.

**Figure 5:**
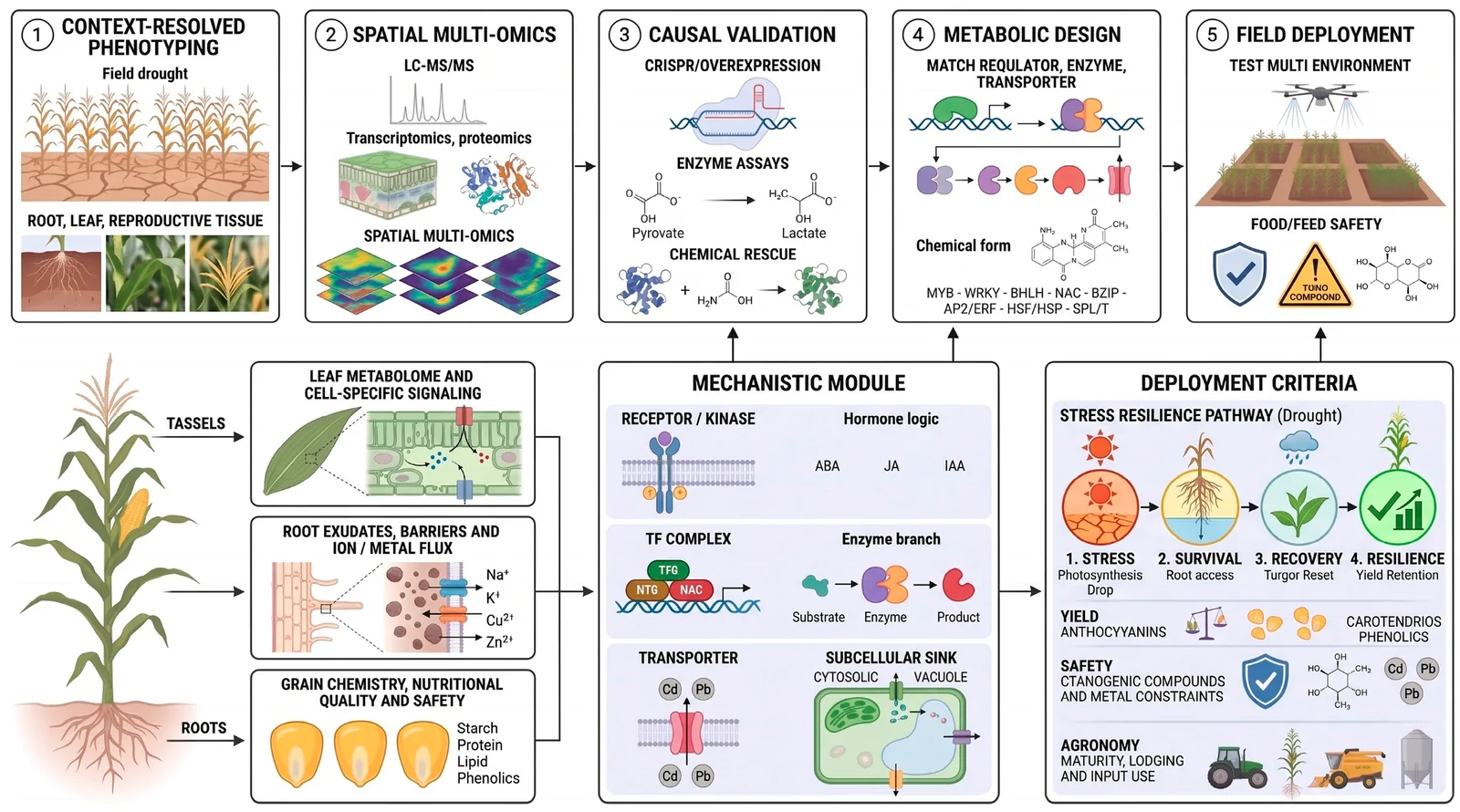
Experimental pipeline for translating secondary metabolite discovery into cereal improvement. Targeted, widely targeted, untargeted, spatial, and flux-based analyses identify candidate regulator–enzyme–metabolite–transport modules. Analytical quality control and annotation confidence determine the reliability of metabolite discovery [13]. Temporal analysis, genetic perturbation, biochemical assays, isotope tracing, localization, complementation, and chemical rescue test causal function [18]. Validated modules can inform metabolite-assisted selection, genomic prediction, promoter editing, and metabolic engineering. Multi-environment evaluation must assess recovery, yield, grain quality, food or feed safety, and growth–defence trade-offs before a pathway is prioritized for breeding.

**Table 1:** Molecular control modules that connect abiotic-stress signaling with secondary-metabolite functions in cereals.

Control Module	Representative Components	Principal Pathway Targets	Subcellular Site	Mechanistic Function	Evidence Level	Representative Literature
Stress perception	RLKs/RLPs; OSCA; CNGCs; mechanosensitive channels	RBOH activation; Ca^2+^ signatures; phospholipid signals	PM/Chl/Mito	Converts hydraulic, ionic, thermal, oxygen and metal perturbations into coded signals	Causal	[29,30,31]
Kinase decoding	CDPK/CPK; CBL–CIPK; SnRK2; MAPK modules	Ion channels; TF phosphorylation; RBOH feedback	Cyt/Nuc/PM	Controls signal duration, amplification and specificity	Causal	[32,33]
Hormonal integration	ABA; JA; SA; ethylene; auxin; BR	Osmotic adjustment; defence allocation; phenology	Multiple	Coordinates growth–defence balance and recovery	Causal	[34,35]
MYB–bHLH–WD40	MYB, bHLH and WD40 complexes	PAL, CHS, F3H, FLS, DFR, ANS, UGTs	Nuc/ER	Controls flavonoid, anthocyanin and phenolic branch flux	Causal	[36,37]
WRKY/NAC/ERF/HSF	WRKY, NAC, ERF/AP2, HSF and bZIP families	Phenylpropanoids, lignin, hypoxia and heat-response genes	Nuc	Links redox and hormone signals with structural and stress-specific responses	Associative	[38,39]
Phenylpropanoid entry	PAL, C4H, 4CL, HCT	Hydroxycinnamates and precursor supply	Cyt/ER	Antioxidant chemistry, signaling and pathway allocation	Causal	[8,40]
Flavonoid branch	CHS, CHI, F3H, FLS, DFR, ANS	Flavones, flavonols, anthocyanins and proanthocyanidins	ER/Cyt/Vac/Chl	ROS buffering, UV and membrane protection, signaling	Causal/Associative	[41,42,43]
Lignin branch	CCR, CAD, COMT, peroxidases, laccases	Monolignols, lignin and ferulate cross-links	Cyt/Apo/CW	Root barriers, mechanical strength and restricted ion movement	Causal/Associative	[39,44]
Carotenoid branch	DXS, DXR, PSY, PDS, ZDS, LCYB	Carotenes, xanthophylls and ABA precursors	Plastid/Thyl	Energy dissipation, ROS quenching and hormone precursor supply	Causal/Associative	[37,45]
Chemical modification	UGTs, GSTs, methyl- and acyltransferases	Glycosides, methylated and acylated products	Cyt/Golgi	Controls solubility, toxicity, stability and recognition	Causal/Associative	[37,40]
Transport and storage	MATE, ABC and MFS transporters; GST ligandins	Vacuolar and apoplastic pools	Ton/PM/Ves	Determines chemical form, spatial concentration, detoxification capacity and whether metabolites reach membranes, chloroplasts, vacuoles or cell walls.	Causal/Associative	[8,37,40]
Feedback regulation	Metabolites, ROS, hormones and chromatin	TF activity and enzyme turnover	Nuc/Cyt/Org	Prevents overaccumulation and adjusts flux during recovery	Associative	[31,33]

PM, plasma membrane; Cyt, cytoplasm; Nuc, nucleus; Chl, chloroplast; Mito, mitochondrion; ER, endoplasmic reticulum; Vac, vacuole; Apo, apoplast; CW, cell wall; Thyl, thylakoid; Ton, tonoplast; Ves, vesicles. Causal, supported by genetic or experimental perturbation demonstrating a direct effect on the phenotype; Associative, supported primarily by correlations, genotype comparisons, transcriptomics, metabolomics, multi-omics integration, or pathway enrichment.

**Table 2:** Mechanistically informative cereal studies used to connect molecular regulation, chemistry and stress-related phenotypes.

Crop and Citation	Stress/Design	Tissue and Platform	Metabolites/Regulators	Mechanistic Interpretation	Evidence Level	Physiological or Agronomic Outcome
Rice—[41]	Contrasting drought response	Seedling tissues; transcriptome–metabolome and biochemical assays	Flavonoids, phenolic acids, glutathione; antioxidant enzymes	Coordinated flavonoid and glutathione biosynthesis strengthened enzymatic and non-enzymatic redox buffering	Associative/In vitro	Lower oxidative injury and improved drought tolerance
Foxtail millet—[44]	Booting-stage field drought	Flag leaves; UPLC–MS and RNA-seq; reproductive traits	Phenylpropanoid, monolignols; CCR and related genes	Reproductive-stage drought redirected phenylpropanoid and lignin metabolism while modifying photosynthesis	Associative	Root/leaf acclimation and yield-component protection
Winter wheat—[49]	Drought followed by rehydration	Leaves; metabolomics and transcriptomics	Flavonoids and redox-related genes	Recovery required active flavonoid turnover rather than passive persistence of the drought state	Associative	Improved regrowth and redox recovery
Sorghum—[66]	Genotype comparison under drought	Leaves; integrated transcriptome and metabolome	Flavonoids, phenylpropanoid and glutathione-linked metabolism	Tolerant material showed coordinated pathway activation rather than isolated metabolite accumulation	Associative	Antioxidant defense and maintenance of growth
[67]	Drought-tolerant and sensitive genotypes	Leaves and roots; proteomics and metabolomics	Phenolic, flavonoids, amino-acid/redox context	Organ-specific metabolic and protein responses differentiated tolerance mechanisms	Associative	Better preservation of root and leaf function
Barley—[47]	Field drought across genotypes	Leaves; targeted phenolic and antioxidant assays	Phenolic compounds and antioxidant enzymes	Phenolic profiles interacted with enzymatic defense and genotype	Associative	Reduced oxidative damage under field drought
Synthetic wheat—[42]	Salinity comparison with parents	Leaves/roots; physiological and metabolite analyses	Flavonoids, phenolic and lignin-related traits	Tolerance inherited a flavonoid-centered redox and structural strategy from the tolerant parent	Associative	Improved Na^+^/K^+^ balance and growth
Rice—[52]	Salt-tolerant versus sensitive varieties	Leaves; metabolomics	Serotonin, gentisic acid and related metabolites	Tolerance involved selective aromatic-metabolite accumulation rather than a universal total-phenolic increase	Associative	Reduced oxidative injury
Foxtail millet—[54]	Saline–alkali stress	Seedlings; integrated transcriptome–metabolome	Flavonoids, phenylpropanoid, osmotic and ion-homeostasis pathways	High pH and ionic stress activated interacting metabolic and regulatory networks	Associative	Improved osmotic and ionic adjustment
Rice—[50]	Cultivar-specific salinity exposure	Seedlings; NMR/metabolomics and morph physiology	Phenolic and cultivar-specific metabolite signatures	Distinct cultivars used different chemical and physiological routes to tolerance	Associative	Maintained growth and ion balance
Maize—[53]	Shoot response to salt stress	Shoots; transcriptome–metabolome	Phenylpropanoid, flavonoids and transport-related genes	Salt altered coordinated shoot metabolic and transcriptional modules	Associative	Membrane/redox protection and ion-homeostasis context
Wheat—[43]	Root thermotolerance with multi-omics and ML prioritization	Roots; metabolomics, transcriptomics and physiological assays	Flavonoids and key pathway genes	Cross-platform evidence prioritized flavonoids as heat-responsive effectors	Associative	Improved root thermotolerance
Rice—[37]	OsDUGT1 overexpression/knockout under heat	Leaves and seedlings; LC–MS/MS, transcriptomics, enzyme and survival assays	Flavonoid glycosides; *OsDUGT1*	Genetic perturbation linked glycosylation, flavonoid compartmentation and heat tolerance	Causal	Higher survival and antioxidant capacity
Maize—[38]	Heat response of hybrid ZD309	Leaves; transcriptome–metabolome	Carotenoids, phenolic, hormones and TF networks	Heat tolerance emerged from coordinated regulatory and metabolic modules	Associative	Photosynthetic and cellular protection
Oat—[45]	Contrasting heat response	Leaves; transcriptome, metabolome and lipidome	Flavonoids, phenylpropanoid and lipid-associated metabolites	Metabolic protection was coupled to membrane-lipid remodeling	Associative	Improved thermal acclimation
Barley—[39]	Waterlogging time course	Roots; transcriptome–metabolome	Phenylpropanoid, flavonoids, hypoxia and fermentation genes	Hypoxia signaling interacted with structural and antioxidant metabolism	Associative	Root survival and waterlogging tolerance
Sorghum—[59]	Cadmium stress	Roots; transcriptome–metabolome	Flavonoid pathway genes and metabolites	Flavonoid reprogramming was integrated with root detoxification and redox control	Associative	Reduced Cd injury and improved root performance
Cross-cereal synthesis—[68]	Multiple abiotic stresses	Multi-omics review and framework	Conserved TF–enzyme–metabolite modules	Integration is strongest when omics associations are tested by perturbation and biochemical validation	Associative	Translational prioritization across environments

Evidence levels: Causal, supported by genetic or experimental perturbation demonstrating a direct effect on the phenotype; Associative, supported primarily by correlations, genotype comparisons, transcriptomics, metabolomics, multi-omics integration, or pathway enrichment; In vitro, supported by biochemical or molecular assays conducted outside the intact plant. Studies containing more than one evidence type are classified according to the strongest evidence directly supporting the stated mechanism.

**Table 3:** Translational roadmap for converting secondary-metabolite mechanisms into deployable cereal stress-resilience targets.

Translational Target	Mechanistic Requirement	Recommended Validation	Expected Benefit	Principal Risk or Trade-Off	Representative Literature
Flavonoid-regulatory complexes	Define MYB–bHLH–WD40 targets, tissue and chemical products	Promoter editing; allelic series; LC–MS/MS; ROS imaging; Field reproductive stage validation	Redox buffering and membrane/photosystem protection	Carbon cost, pigmentation or altered development	[36,37,41]
Root lignin/suberin modules	Separate barrier deposition from general growth inhibition	Root imaging; lignin chemistry; hydraulic and ion-flux assays; Field reproductive stage validation	Reduced water/ion loss and metal translocation	Restricted elongation, forage quality and nutrient uptake	[39,44]
Carotenoid/xanthophyll control	Coordinate PSY/PDS/ZDS flux with thylakoid demand and cleavage	Pigment profiling; chlorophyll fluorescence; ABA measurements; Field reproductive stage validation	Photoprotection and hormone precursor supply	Depletion of pigments by excessive cleavage	[37,45]
Flavonoid glycosylation and transport	Pair UGT activity with GST/MATE/ABC transport and vacuolar pH	Genetic perturbation; enzyme assay; subcellular imaging; Field reproductive stage validation	Stabilized metabolites and reduced aglycone toxicity	Sequestration may remove active compounds from target sites	[37]
Dhurrin/cyanogenic metabolism	Resolve synthesis, storage, turnover and nitrogen recycling	Tissue-specific CYP/UGT editing; cyanide and yield assays; Field reproductive stage validation	Defence and possible nitrogen remobilization	Food/feed toxicity and stress-induced overaccumulation	[87]
Benzoxazinoid modules	Distinguish constitutive storage from stress-triggered activation	Bx-gene alleles; exudate and microbiome profiling; Field reproductive stage validation	Root defence and ecological signaling	Allelopathy and context-dependent crop interactions	[88]
Phenolic metal-binding systems	Demonstrate direct chelation or wall immobilization	Speciation, ionomics, imaging mass spectrometry and transport assays; Field reproductive stage validation	Reduced free-ion activity and grain contamination	Metal–metabolite complexes may remain mobile	[59]
Hypoxia/reoxygenation modules	Separate low-O_2_ acclimation from oxidative recovery	Time-course ERF-VII, fermentation, ROS and wall measurements; Field reproductive stage validation	Root survival and rapid recovery	Fermentation costs and delayed growth	[39]
Stress-inducible promoters	Restrict pathway activation to the relevant tissue and window	Cis-regulatory editing; reporter lines; field stress timing; Field reproductive stage validation	Lower constitutive growth penalty	Promoter response may vary among environments	[68]
Metabolite-assisted breeding	Use chemically identified, heritable and stable markers	Multi-environment QTL/GWAS; genomic prediction; external validation; Field reproductive stage validation	Improved selection for complex tolerance modules	Population specificity and analytical batch effects	[89]
Recovery-focused phenotyping	Measure repair and remobilization after stress release	Repeated phenotyping; redox and metabolite time courses; Field reproductive stage validation	Selection for resilience rather than survival alone	Recovery benefit may trade off with grain filling	[49]
Quality and safety co-optimization	Measure nutritional metabolites and harmful compounds together	Grain metabolomics; processing and feeding assessments; Field reproductive stage validation	Stress tolerance with improved grain value	Cyanogenic, alkaloid or contaminant accumulation	[37,45]

## Data Availability

This manuscript is a review article and does not report primary data. All data referenced herein are available from the cited published sources.
